# Intracranial EEG recording in autoimmune epilepsy dataset

**DOI:** 10.1016/j.dib.2019.104191

**Published:** 2019-06-27

**Authors:** Lisa Gillinder, Sasha Dionisio

**Affiliations:** Mater Advanced Epilepsy Unit, Mater Hospital, Brisbane, Australia

**Keywords:** Autoimmune epilepsy, Stereo-electroencephalography (SEEG), Intracranial recordings

## Abstract

The appearance of seizures in autoimmune epilepsy on intracranial recordings has not been previously demonstrated. The following data shows a multifocal epilepsy in a patient with seronegative autoimmune epilepsy (reported here; “Electroclinical Insights into Autoimmune Epilepsy”, Gillinder, 2019). Independent seizures were seen to arise from 5 separate foci. These all began with slow repetitive spiking in a highly restricted area. Only after many minutes would this activity spread to other regions. Despite arising from different locations, all foci affected the posterior insula resulting in clinical symptoms.

**Table:** Specifications table

Subject area	*Biology*
More specific subject area	*Epilepsy*
Type of data	*Image (SEEG) presented in bipolar montage*
How data was acquired	*Intracranial depth electrode implantation (using DIXI electrodes - DIXI medical, Besançon, France)*
Data format	*Filtered (stored as a modified Brain electrical source analysis (BESA) file, can be exported as American Standard Code for Information Interchange (ASCII), Comma separated values (CSV) or European data format (EDF) format).*
Experimental factors	*Stereotactic-EEG (SEEG) data was acquired on a Nihon Khoden (NK) EEG-1200 system using* 1KHz*-sampling rate and 16-bit* analogue to digital conversion (*ADC's) and physical highpass filters of 0.*08Hz *applied. A bipolar referencing montage (between adjacent SEEG electrode contacts was used for interpretation, as is common in clinical practice**[2]**. SEEG data acquired while off anti-epileptic medications before and after Intravenous Immunoglobulin (IVIg) and Methylprednisone is presented and compared.*
Experimental features	*Stereo-electroencephalography (SEEG) implantation*
Data source location	*Mater Centre for Neurosciences, South Brisbane, Queensland, Australia*
Data accessibility	*Raw data available at Mater Centre for Neurosciences (Raw data can be provided upon request to the corresponding author).*
Related research article	L. Gillinder, J. Papacostas, and S. Dionisio, *Electroclinical insights into autoimmune epilepsy.* J Neuroimmunol, 2019.**330**: p. 44–47 [1].

**Table:** 

**Value of the data** • Multifocal seizure onset and slow repetitive spiking were features in this data which may be used to identify cases of autoimmune epilepsy. • This epilepsy strongly involved the perisylvian regions, particularly the posterior insula and this data may be useful to consider which patients should undergo autoimmune screening [3]. • This dataset provides insights into the pattern of seizure propagation in autoimmune epilepsy which may be the focus of future research into the mechanisms of autoimmune epileptogenesis. • These features are unique, providing potential insights into the nature of the pathogenesis of autoimmune epilepsy and their presence should alert clinicians to the possibility of an autoimmune aetiology.

## 1 Data

This SEEG evaluation demonstrated a multifocal epilepsy in the right hemisphere of a patient with seronegative autoimmune epilepsy. Independent seizures were seen to arise from the right posterior insula, temporal operculum, posterior middle temporal gyrus (MTG) and superior temporal gyrus (SMG). These all began with slow repetitive spiking in a highly restricted area. After many minutes this would spread to other regions. Despite arising from different locations, all foci spread to affect the posterior insula resulting in clinical symptoms. Even the seizures arising from the posterior insula began with slow repetitive spiking, evolving over many minutes, and did not involve fast activity. The seizures and epileptic activity resolved after administration of immunotherapy (IT).

## 2 Experimental design, materials and methods

**Image 1:**
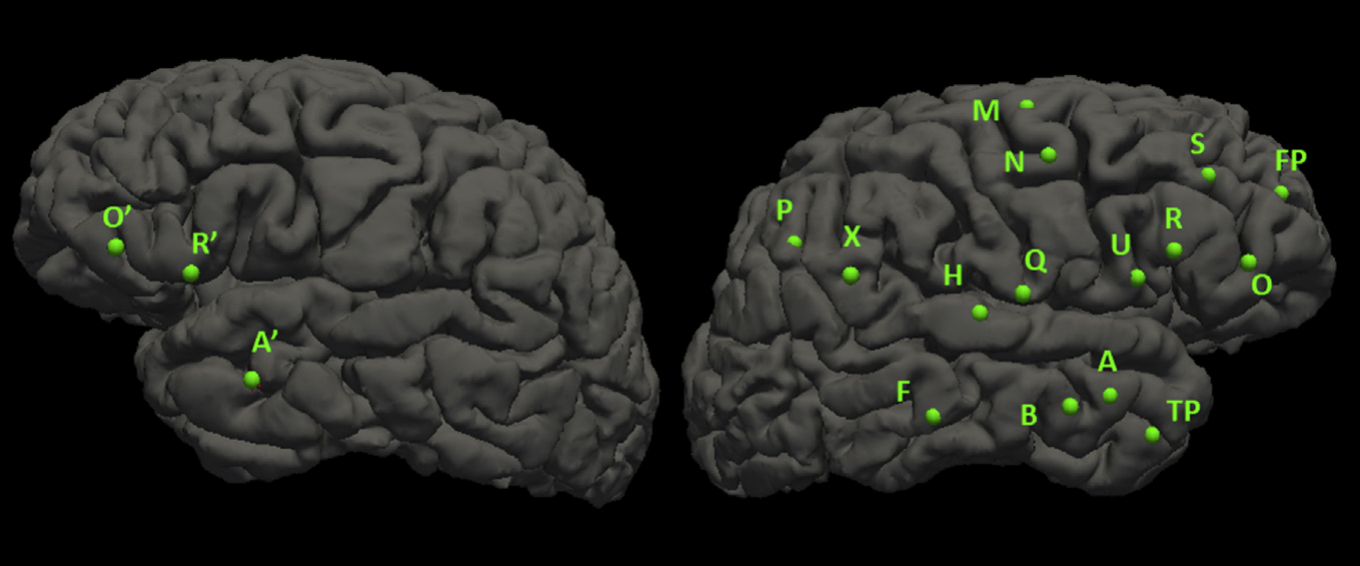
Surface map of implanted electrodes, reconstructed from raw MRI data. Implantation was heavily weighted to the right hemisphere based on the Video EEG evaluation which only demonstrated right sided seizures.

**Image 2:**
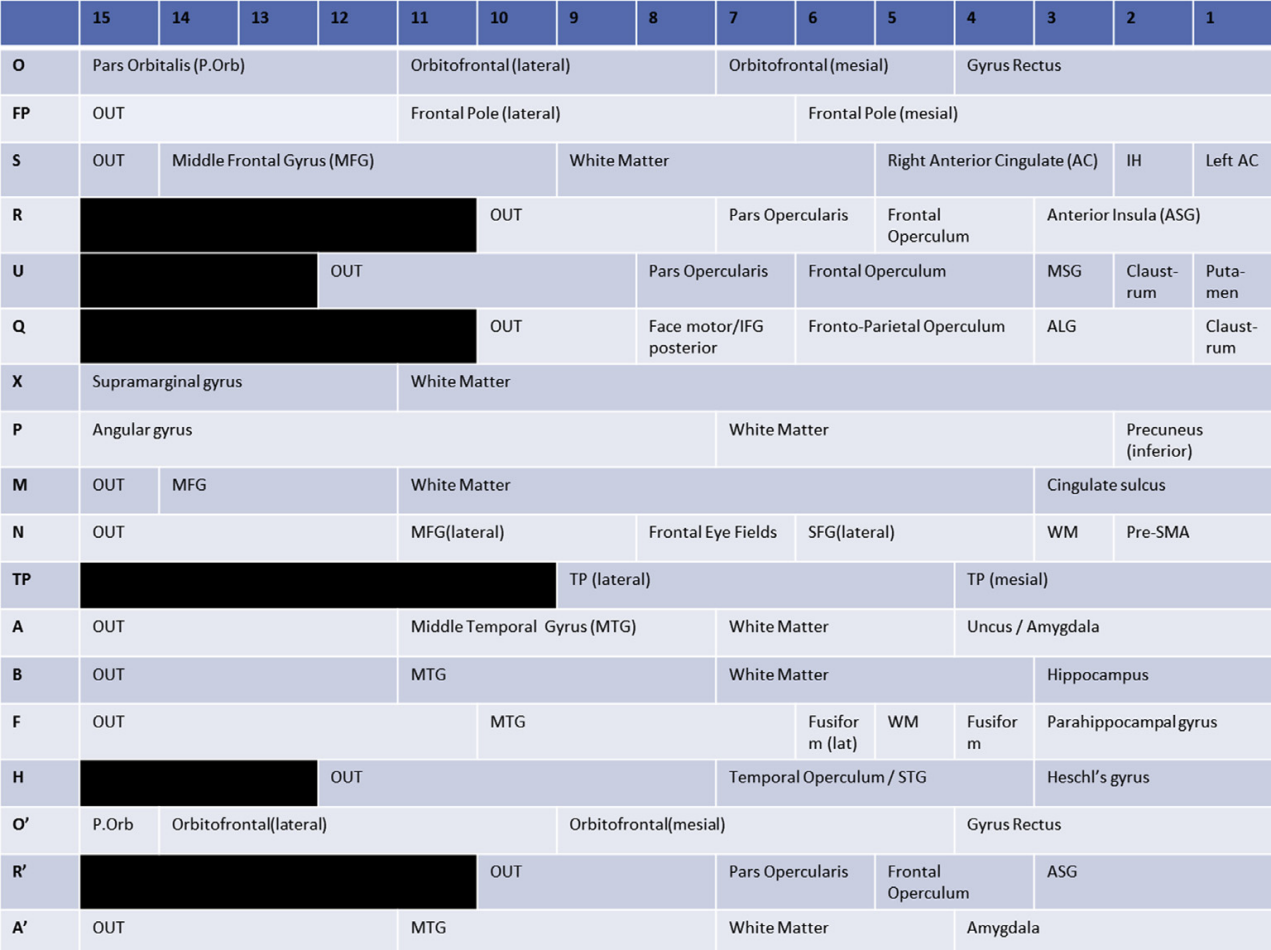
Contact map of implanted electrodes outlining anatomical locations of each electrode contact. Alphanumeric codes in the following image descriptions are taken from this map.

**Image 3:**
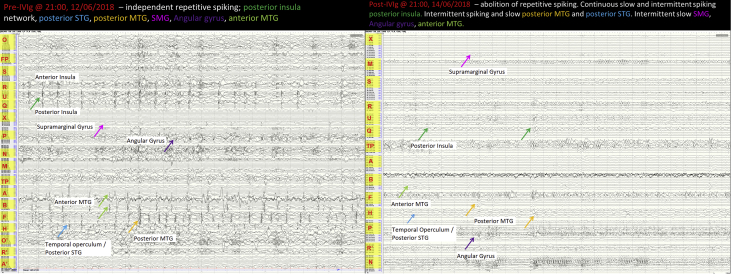
A: Interictal changes during implantation and off anti-seizure drugs. Multi-regional independent spiking was seen in right posterior insula, temporal operculum/posterior STG, posterior MTG, Supramarginal gyrus and anterior MTG. B: 24 hours after administration of intravenous immunoglobulin and methylprednisone the spiking seen previouly had resolved. This occurred while the patient remained off anti-seizure medications.

**Image 4:**
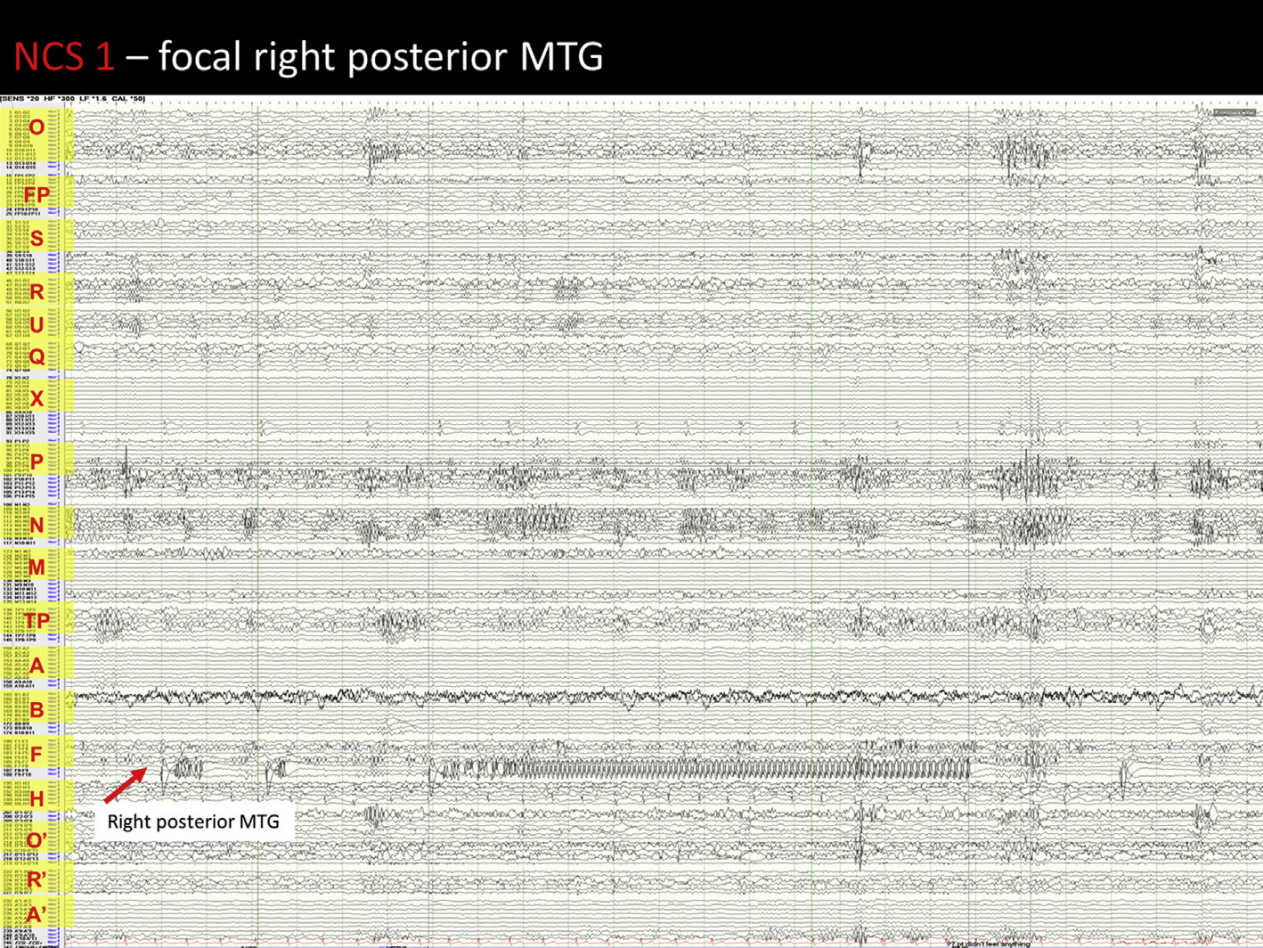
NCS 1 – Right posterior MTG seizure: Prior to seizure onset there is a significant increase in the spiking seen in the right posterior MTG (F 7–9). At seizure onset there is a large amplitude spike in this region with an associated DC shift that is highly localised and does not affect other regions. This is followed by rapid repetitive spiking which resembles the preictal activity, however is sustained and continues in a semirhythmic pattern for 2 seconds. There is then a sudden increase in the rhythmicity of the repetitive spiking and a reduction in frequency. The activity remains localised to the posterior middle temporal gyrus (F7-9). This pattern continues until seizure end. Post ictally there is slowing localised to the right posterior MTG, and within 4 seconds interictal spiking resumes.

**Image 5:**
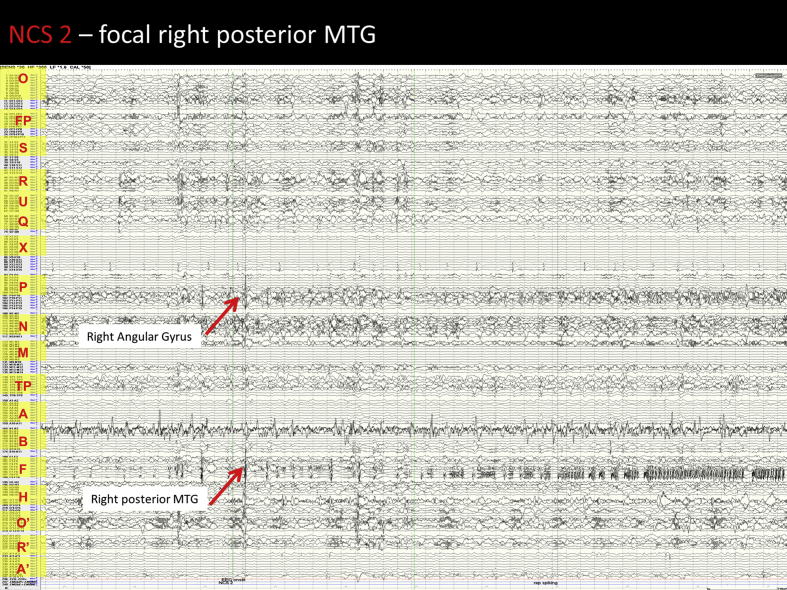
NCS 2 – Right posterior MTG seizure: Prior to seizure onset there are spikes which are seen maximally in the right posterior middle temporal gyrus (F 7–9) and also affect the right mesial angular gyrus (P9-12). At seizure onset there is a large spike with DC offset affecting the right posterior middle temporal gyrus and lateral right fusiform gyrus (F5-9). This is followed by low amplitude rapid repetitive spiking seen maximally in the lateral fusiform gyrus for 1 second. There are then further larger spikes with interspersed low amplitude rapid repetitive spikes in a repeating pattern for a prolonged period. The repetitive spiking activity becomes progressively more sustained between the larger spikes. During this time the activity in the right mesial angular gyrus is modified but not clearly involved. There is also less pronounced modification of the right posterior superior temporal gyrus. Repetitive low amplitude spiking becomes the dominant activity but remains localised to the right posterior MTG until seizure end. Post ictally there is reduced activity in the right posterior MTG for some time, however interictal spiking returns within 1 min.

**Image 6:**
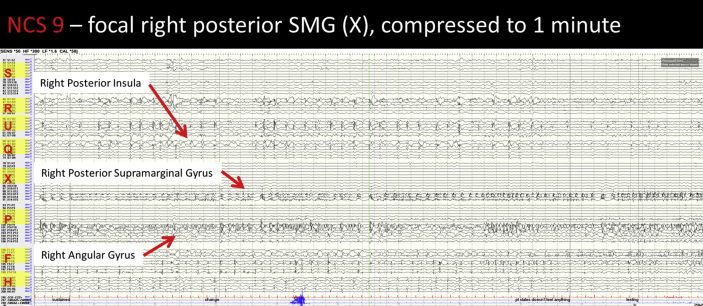
NCS 9 – right supramarginal gyrus seizure: There is an increase in the interictal spiking seen in right supramarginal gyrus (X 12–14) at seizure onset. This then becomes sustained and spreads to affect the right lateral angular gyrus (P 14). The activity also affects the mesial angular gyrus (P 9–13) to a lesser extent. This pattern continues for a prolonged period. As the seizure progresses the activity in the right SMG is less sustained, with periodic bursts of rapid repetitive spiking. This pattern continues until seizure end.

**Image 7:**
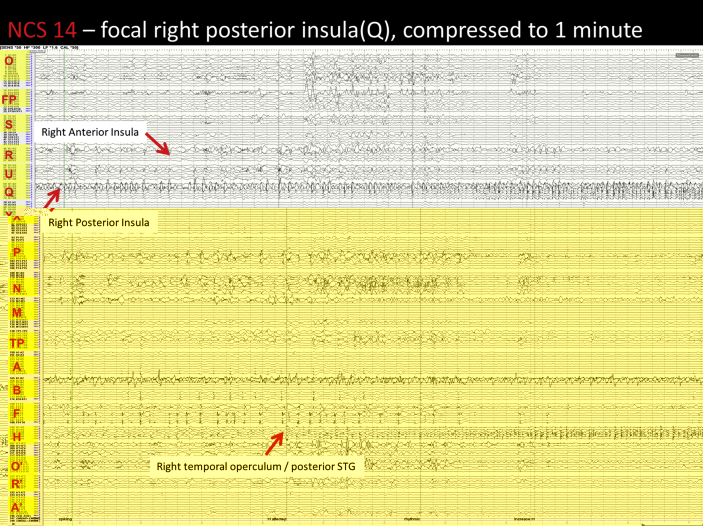
NCS 14 – right posterior insula seizure: Pre ictally there is an increase in the repetitive spiking seen in the right posterior insula (Q2-3) and anterior insula (MSG U 2–4). At seizure onset there is a spike of lower amplitude in the posterior insula which is followed by low amplitude semi rhythmic activity and within 3 seconds this increases in amplitude. The posterior insula activity synchronises the frontoparietal operculum (Q5-7), but to a much lesser degree. The mid cingulate is also affected by a low amplitude rhythmic activity and the right posterior STG become affected a low amplitude repetitive spiking which is synchronised to the activity in the posterior insula. Maximal activity remains in the posterior insula until seizure end. Post ictally there is slowing seen in the posterior insula and frontoparietal operculum. This continues for over 10 minutes.

**Image 8:**
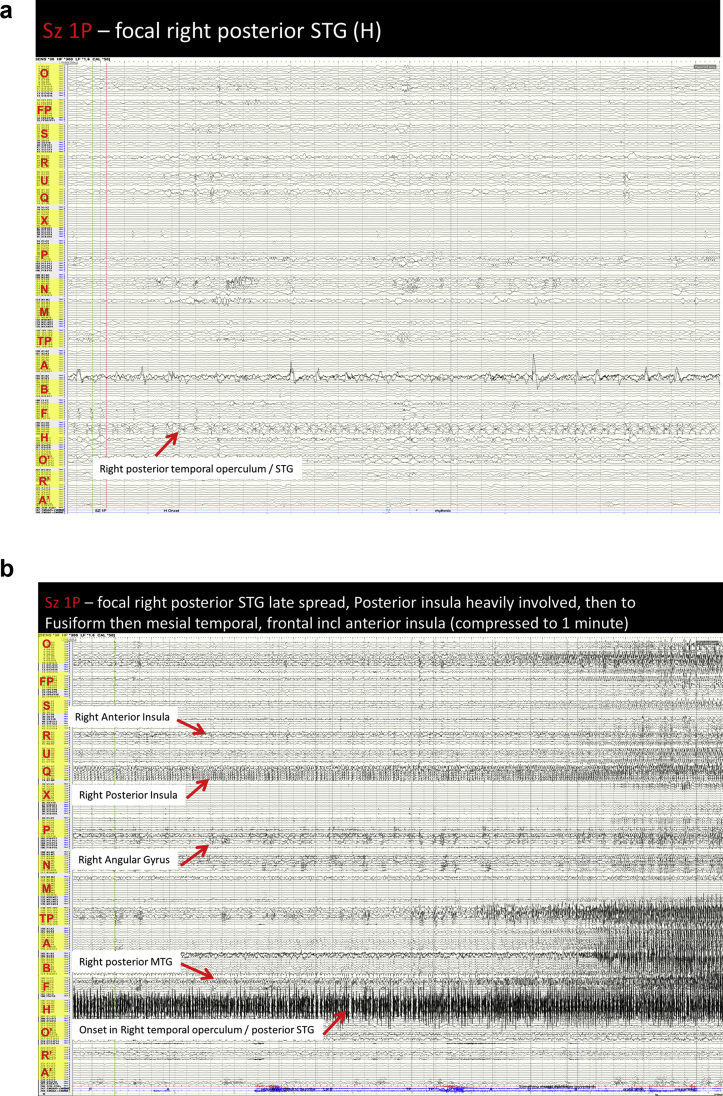
Sz 1P – right temporal operculum/posterior STG seizure: A: Prior to seizure onset there is an increase in the interictal repetitive spiking see in the right posterior superior temporal gyrus (H 3–5). At seizure onset there is a semi-rhythmic activity in the right posterior superior temporal gyrus with ongoing spiking. This appears as a spread pattern. While the activity does not affect the right posterior middle temporal gyrus (F 7–9), the interictal activity is modified with a reduction in repetitive spiking. The activity remains localised for a prolonged period, but then synchronisation between the posterior STG and Heschl's gyrus becomes clearer. B: The maximal activity remains in the right posterior STG as repetitive spiking and only after another prolonged period is there involvement of the posterior insula, however this coincides with onset of clinical symptoms. Late seizure spread affects the right frontoparietal operculum, posterior and anterior MTG (F 7–9)(B 7–9)(A 8–11), right fusiform gyrus is modified (F 2–3), right temporal pole (TP 1–5), right amygdala (A 1–2), right orbitofrontal region (O 6–9). Other areas are also affected to a much lesser degree; frontal pole (FP 2), anterior cingulate (S 1–4), anterior insula (R 1–2, U 2–4), posterior cingulate (X 1–2), inferior precuneus (1–2). The left hemisphere is modified but not affected.

**Image 9:**
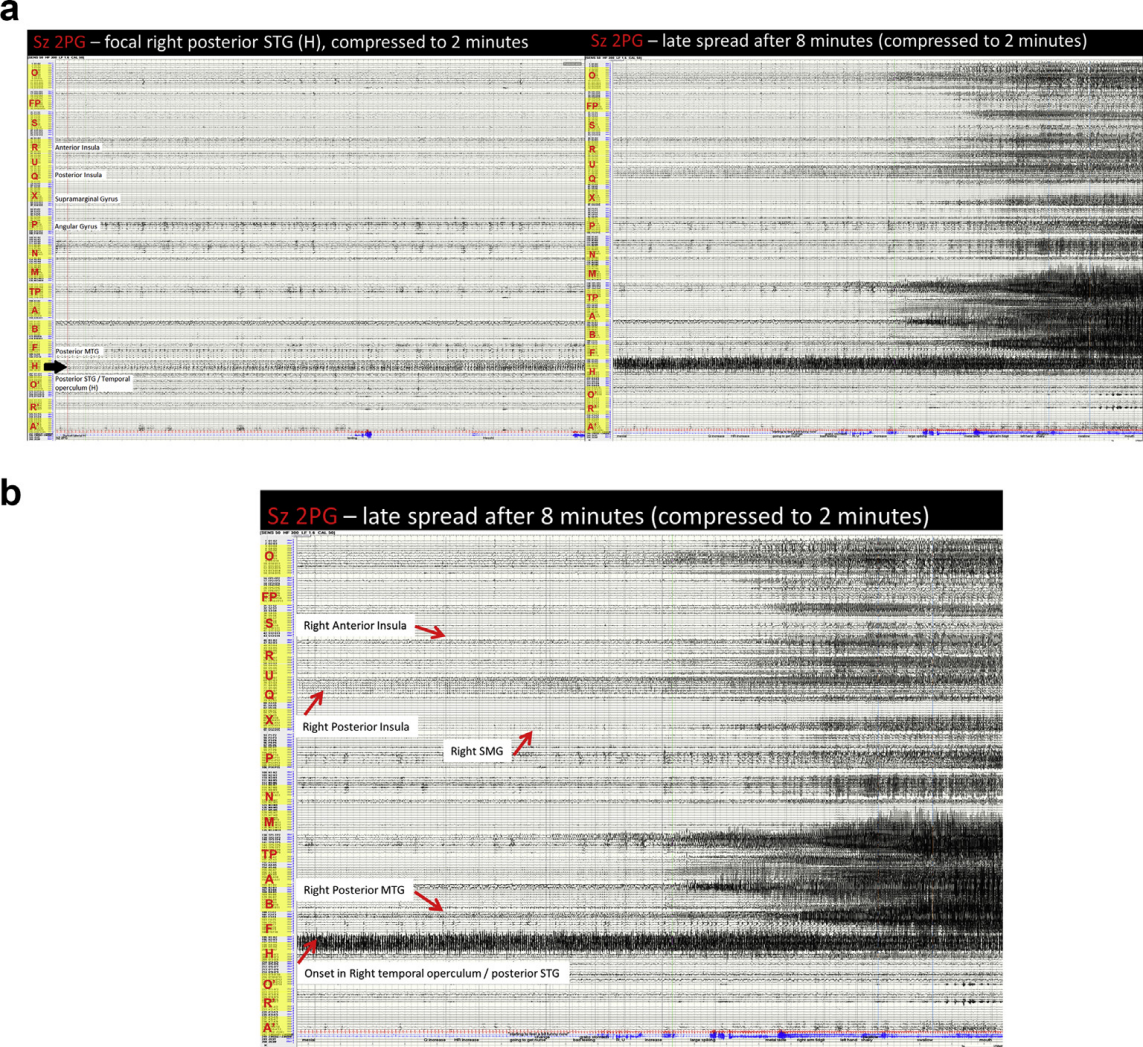
Sz 2PG – right posterior STG seizure: There is an increase in the repetitive spiking seen in the right posterior STG. This continues for a prolonged period without affecting other areas. Repetitive spiking continues in the right posterior MTG and is not affected by the seizure. The activity then spreads mesially to affect Heschl's gyrus, but still remains highly localised. The right posterior insula becomes modified but is not affected. After a period the posterior insula becomes affected by a low amplitude rhythmic activity and this coincides with clinical onset. The anterior insula is modified but not affected. Maximal activity remains in the posterior STG.

**Image 10:**
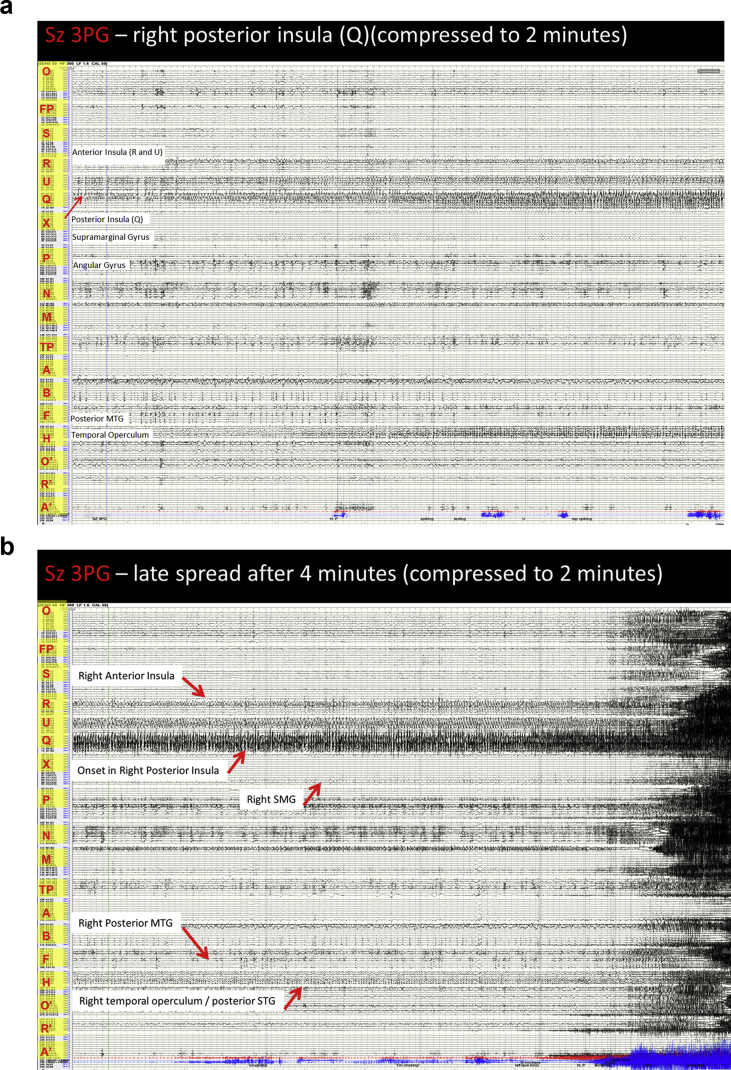
Sz 3PG – right posterior insula seizure: A: At onset the interictal spiking in the posterior insula network ceases and is replaced by moderate amplitude rhythmic slow activity. This modifies the activity in the right anterior insula and within 30 seconds spreads to affect it. It also affects the posterior cingulate (X 1) and supramarginal gyrus (X 12–14) and mid cingulate (M 1–2) at this time, which is much earlier than in the previous seizure. The maximal activity remains in the posterior insula for a prolonged period and evolves into periodic rapid repetitive spiking, which spreads laterally to affect the frontoparietal operculum. B: There is a sudden increase in the frequency of the activity in the posterior insula. This affects the STG to a much lesser extent. The activity spreads to affect the mesial angular gyrus (P 8–9) and SFG (N 3–6) and then moves laterally. Within 2 seconds the anterior cingulate and orbitofrontal regions are also affected. The fusiform gyrus becomes affected and the activity spreads laterally to affect the MTG with repetitive spiking. The temporal pole is also affected and within 4 seconds the hippocampus is also affected. The left orbitofrontal region is also affected. Post ictally there is diffuse cerebral attenuation seen post ictally. Frequencies return first to the left hemisphere, then the right frontal regions. There is prolonged slowing in the posterior insula.

The patient underwent bilateral temporo-perisylvian implantation and was recorded for 5 days off anti-epileptic medications. 18 semi-flexible platinum electrodes were implanted using stereotactic guidance. Each electrode has a diameter of 0.8mm, a contact length of 2mm and an intercontact insulation length of 1.5mm. Data was recorded using the Nihon Khoden (NK) EEG-1200 system with 1KHz-sampling rate and 16-bit ADC's and a physical highpass filter of 0.08Hz applied. The exact positions of the electrodes were verified by visual analysis of a co-registered post implantation computed tomography (CT) with a pre-implantation structural magnetic resonance imaging (MRI), by an epileptologist. Involvement of suspected epileptogenic zone (EZ) locations was confirmed by using cortical stimulation to replicate habitual seizures or evoke typical seizure behaviors. A total of 19 seizures were recorded during the evaluation arising from 5 separate foci within the right hemisphere. Intravenous immunoglobulin and methlyprednisone was administered while the patient remained off anti-epileptic medications which resulted in seizure cessation and normalization of the interictal epileptiform abnormalities [1]. (see Image 1, Image 2, Image 3, Image 4, Image 5, Image 6, Image 7, Image 8, Image 9, Image 10).

## Anatomical abreviations

MTG: Middle temporal gyrus
STG: Superior temporal gyrus
SFG: Superior frontal gyrus
SMG: Supramarginal gyrus
ASG: Anterior short gyrus of the insula
MSG: Middle short gyrus of the insula
